# Periocular triamcinolone acetonide injection for treating polypoidal choroidal vasculopathy concurrent with hemorrhagic retinal detachment

**DOI:** 10.1097/MD.0000000000012464

**Published:** 2018-09-28

**Authors:** Kaiyan Zhang, Yingying Chen, Xuyang Sun, Qionglei Zhong, Lin Lin, Yuan Gao, Fanlin Hong

**Affiliations:** Department of Ophthalmology, Hainan General Hospital, Haikou, China.

**Keywords:** hemorrhagic retinal detachment, periocular injection, polypoidal choroidal vasculopathy, triamcinolone acetonide

## Abstract

To investigate the clinical efficiency of periocular triamcinolone acetonide (TA) injection for treating polypoidal choroidal vasculopathy (PCV) concurrent with hemorrhagic retinal detachment (HRD).

Twenty-two cases confirmed with PCV concurrent with HRD characterized by massive subretinal hemorrhage and exudation presented to our department from January 2015 to May 2017 were included in this study. The initial vision varied from counting finger to 0.2. All cases were randomly divided into TA group (n = 12), which received periocular TA injection per month, and anti-VEGF group (n = 10), which were treated by anti-VEGF intravitreous injection per month. The patients were followed up for 6 months, in which fundus examination and visual acuity along with optical coherence tomography (OCT) were carried out.

The treatment effect is divided into the following categories. Cure was defined as the elimination of subretinal hemorrhage and exudation accompanied by retinal edema and choroidal neovascularization (CNV) extinction and rise of visual acuity. Improvement was characterized by alleviation of subretinal hemorrhage and exudation accompanied by retinal edema and CNV reduction and rise of visual acuity. Ineffective means remained subretinal hemorrhage and exudation in fundus and no improvement of visual acuity, and polypoid lesions in OCT images. Among the 12 cases in TA group, 1 case was treated by periocular injection of TA twice, and 11 cases were treated by 3 times injection. After that, 3 cases (25%) were cured, 8 cases (66.7%) got improvement, and only 1 case (8.3%) showed no response. Although among 10 cases in the anti-VEGF group, 3 cases were treated by anti-VEGF intravitreous injection twice. Seven cases were treated by 3 times injection. After that, 4 cases (40%) got improvement, and the other 6 case (60%) showed no response. All patients showed no recurrence in the 6-month follow-up. No complications were noticed under periocular injection or intravitreous injection.

Periocular TA injection is effective for treating PCV concurrent with HRD.

## 1 Introduction

Polypoidal choroidal vasculopathy (PCV) refers to a retinal disorder involving choroidal vasculature featured by presence of polypoidal lesions beneath the retina combined with branching vascular network, as well as vasodilatation in the choroid.^[1]^ Most of PCV cases presented subretinal hemorrhage in posterior pole, serous or hemorrhagic pigment or neural epithelium, detachment, as well as lipid deposition. For these patients, the findings of fluorescein angiography are not specific. In clinical settings, indocyanine green angiography is the golden standard for the diagnosis of PCV, which are mainly featured by alternations in choroid vessels, multiple polypoidal dilatation, bulging lesions, increased staining, and dye leakage at the advanced stage. The most common optical coherence tomography (OCT) findings for PCV include double track sign, pigment epithelium detachment, choroidal thickening, and local choroidal excavation. Presence of intraretinal and subretinal fluid is the landmark for active exudation, which is usually associated with the formation of exudate in fluorescein angiography. Meanwhile, OCT contributes to the monitoring of pigment epithelium detachment and their changes after treatment.^[2]^

Currently, the treatment of PCV is highly relied on the photodynamic therapy (PDT),^[3]^ anti-vascular endothelial growth factor (VEGF),^[4]^ as well as the combination of PDT and anti-VEGF therapy.^[5]^ PDT was reported to induce subretinal hemorrhage.^[6]^ Besides, many patients showed a higher recurrence after PDT.^[7]^ To date, increasing evidence supports that anti-VEGF therapy is effective for the treatment of PCV^[4]^; however, a higher treatment risk may present in the presence of repeated anti-VEGF therapy.^[8]^ The treatment efficiency is still far from satisfaction, especially for those with massive subretinal hemorrhage and exudation. What is more, the treatment cost is expensive for such combination, which hampers its application worldwide.

Triamcinolone acetonide (TA), a synthetic glucocorticosteroid with immunosuppressive and antiinflammatory activity, has been commonly used for treating choroidal neovascularization (CNV) through intravitreal injection.^[9]^ In our previous experiences, periocular injection of TA contributed to amazing recovery of PCV patients concurrent with hemorrhagic retinal detachment (HRD), which was manifested by obvious attenuation in the subretinal hemorrhage and exudation, as well as increase in the visual acuity. Compared with the previous literatures on anti-VEGF therapy, no such efficiency has been obtained. To further approve our observation, PCV patients concurrent with HRD were randomly divided into 2 groups receiving periocular injection of TA and intravitreal injection of anti-VEGF, respectively. We aim to investigate the treatment efficiency of TA and anti-VEGF for treating PCV combined with HRD.

## 2 Materials and methods

### 2.1 Patients

Initial PCV patients complaining of decreased visual acuity in the recent 3 months presented to our department from January 2015 to May 2017 were included in this study. PCV was confirmed using indocyanine green angiography and OCT. The patients showed HRD in fundus oculi with massive subretinal hemorrhage (≥2 disc diameter) and yellow exudate. The exclusion criteria were as follows: those with ocular trauma, ocular surgery, choroid cancer, retinal angioma, hematopathy, as well as other systemic diseases. Finally, 22 cases (male: 14, female: 8, age: 52–78 years) were confirmed with PCV concurrent with HRD symptoms of massive subretinal hemorrhage (≥2 disc diameters) and yellow exudation. Twenty-two cases were randomly divided into TA group (n = 12) and anti-VEGF group (n = 10).

### 2.2 Ethical approval

Each patient signed the informed consent. The study protocols were approved by the Ethical Committee of Hainan General Hospital.

### 2.3 Methods

TA (40 mg, Kenacort-A, Jida Pharmac, Kunming, China) was administrated via periocular injection into the position that was 1.5 cm into the orbit along the orbital margin of the lower eyelid. TA was given in presence of no blood in the pumpback after insertion. Anti-VEGF therapy (0.05 mL, Lucentis, Novartis AG, CA) was administrated via intravitreous injection. Three days before treatment, the patients were treated using Ofloxacin eye drop with a frequency of 4 times per day. After surface anesthesia using Proparacaine Hydrochloride Eye Drops, the conjunctival sac was washed for 90 seconds. Then eyeball wall puncture was given followed by anti-VEGF injection. Each patient received 1 injection per month for 3 months. The patients were followed up once per month after injection until 6 months, during which fundus oculi and visual acuity determination were given. Besides, OCT was carried out, together with observation of the massive subretinal hemorrhage and yellow exudation.

The treatment outcome was divided into cure, improvement, and ineffective. Cure was defined as elimination of the subretinal hemorrhage and yellow exudation, elimination of CNV and retinal edema by OCT, as well as increased visual acuity. Improvement was defined as reduction of subretinal hemorrhage and yellow exudation, decrease of CNV and retinal edema by OCT, as well as increased visual acuity. Ineffective was defined with no treatment response manifested by no or tiny changes in the subretinal hemorrhage and yellow exudation, as well as CNV and retinal edema, and visual acuity. Cure and improvement are defined as effective.

### 2.4 Statistical analysis

SPSS22 software was used for the data analysis. The visual acuity was displayed by mean ± standard deviation. The inter-group comparison was performed using analysis of variance for repeated data. *P* < .05 was considered to be statistically significant.

## 3 Results

The treatment outcomes were categorized into the following categories: cure was defined as elimination of subretinal hemorrhage and exudation accompanied by retinal edema, CNV extinction, and rise of visual acuity; improvement was characterized by alleviation of subretinal hemorrhage and exudation accompanied by retinal edema, CNV reduction, and rise of visual acuity; and ineffective means remained subretinal hemorrhage and exudation, and no improvement of visual acuity, polypoidal lesions, as well as no changes in the retinal edema.

The visual acuity before treatment was 0.07 ± 0.05 for each group (*P* = .758). One month after treatment, the visual acuity in the TA group showed no significant differences compared with that of the anti-VEGF group (0.18 ± 0.11 vs 0.12 ± 0.10, *P* = .213). Significant increase was noticed in the visual acuity in TA group compared with that of the anti-VEGF group at month 2 (0.24 ± 0.12 vs 0.13 ± 0.10, *P* = .037) and month 3 (0.35 ± 0.20 vs 0.16 ± 0.12, *P* = .02, Table 1), respectively.

**Table 1:**
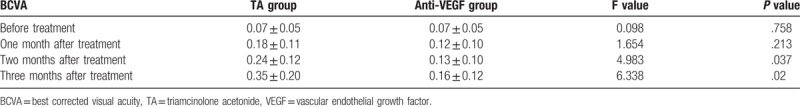
Comparison of visual acuity between 2 groups.

Among the 12 cases in TA group, 1 case was cured after the injection twice, and the other 11 cases received the injection thrice. Finally, 3 cases (25%) showed “cure,” 8 (66.7%) showed “improvement,” and 1 (8.3%) showed “ineffective.”

Among the 10 cases in the anti-VEGF group, 3 cases gave up the treatment after injection twice for the reason of completely ineffective (1 among which vitreous hemorrhage happened). The other 7 cases received injection thrice. Finally, 6 of 12 were improved (60%), 4 of 12 were ineffective (40%). The effective rate is 91.7% in TA group, while that of anti-VEGF group was 60%.

**Figure 1:**
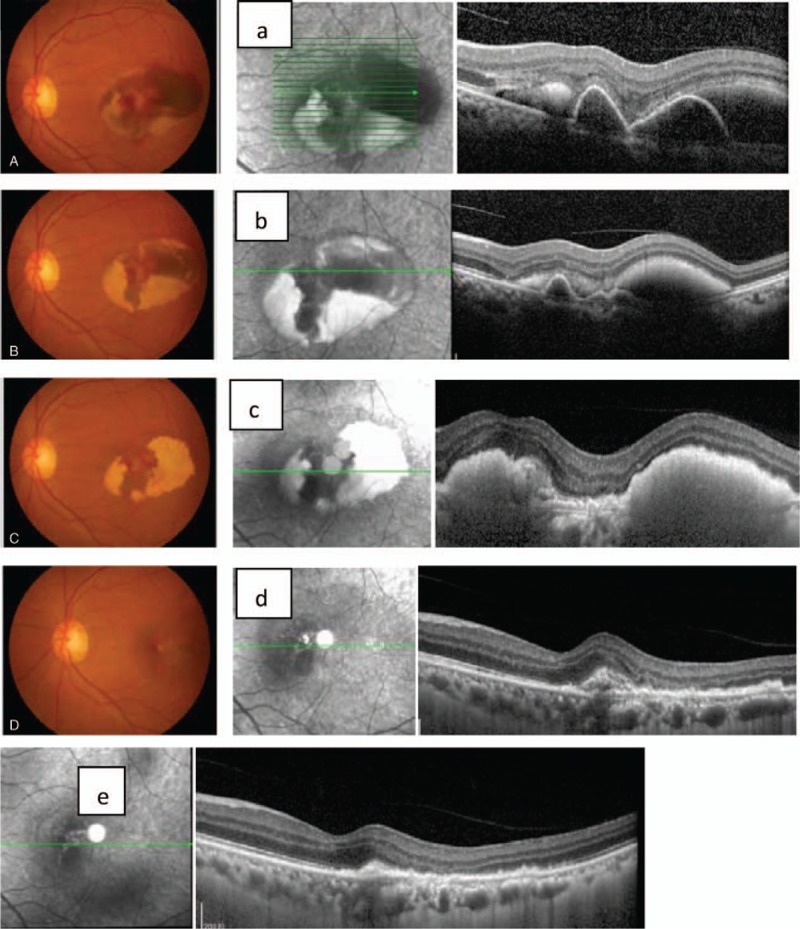
Color fundus photographs and the corresponding period OCT images of PCV patients concurrent with HRD before and after periocular TA injection thrice. (A) Baseline level, the BCVA was 0.2. Massive subretinal hemorrhage in macular. (a) The finger like protrusion of the pigment epithelium and the detachment of the nerve epithelium. (B) Posttreatment 1 month after the 1st time injection. The majority of the subretinal hemorrhage was partly absorbed. Accumulation of yellow-white deposition was noticed. (b) Pigment epithelium protuberant reduced. (C) Posttreatment 1 month after injection twice. The majority of the subretinal hemorrhage was further decreased, while more yellow-white deposition was noticed. (c) The finger like protrusion of the pigment epithelium fade away. (D) Posttreatment 1 month after injection thrice. Subretinal hemorrhage was completely absorbed, and the accumulation of yellow-white deposition was almost eliminated. (d) Only small pigment epithelium protuberances. (e) OCT image of 2 months after the 3rd injection, the pigment epithelium and neuroepithelial detachment almost vanished. The BCVA is 0.7. BCVA = best corrected visual acuity, HRD = hemorrhagic retinal detachment, OCT = optical coherence tomography, PCV = polypoidal choroidal vasculopathy, TA = triamcinolone acetonide.

All the cases showed no recurrence in the 6-month follow-up. No cases showed complications with periocular injection or intraocular injection (Fig. 1).

## 4 Discussion

Most of the PCV patients show subretinal hemorrhage, hemorrhagic or serous pigment epithelium, or neuroepithelial detachment, as well as lipid deposition. The typical pathological features of PCV are presence of choroidal vascular hyalinization, and that massive plasma or fibrin effusion, which is relevant to massive subretinal hemorrhage and exudation. The priority of PCV management is to attenuate the hemorrhage and exudation.

The mechanism of PCV is still not well defined. Nowadays, accumulating evidence indicates that inflammatory factors (e.g., IL-23, C-reactive protein, MCP-1, and tumor necrosis factor-α) were detected in the aqueous humor of the PCV patients.^[10–12]^ Besides, the IL-1β expression was up-regulated in the vitreous body.^[13]^ These aspects demonstrate that PCV may be a type of inflammatory disorder, and the inflammatory factors could interrupt the blood-retinal barrier, which then lead to vascular exudation. On this basis, inflammatory inhibition may serve as a treatment option for treating retinal vessel exudation induced subretinal hemorrhage and exudation, despite the fact that anti-VEGF therapy is a new hot spot nowadays.

Corticosteroids, with anti-inflammatory, anti-hyperplasia, anti-exudation, and immunosuppressive effects, have been preferred for treating ocular inflammation. As systemic administration of corticosteroids may induce severe side effects, local administration of corticosteroids (e.g., sub-conjunctival, intravitreous, and periglomerular injection) have been frequently used for the treatment of ocular posterior inflammation.^[14]^ To date, sub-conjunctival corticosteroid injection is mainly used for treating ocular anterior inflammation, which may induce some complications such as conjunctival ulcer, ptosis, conjunctival congestion, and mydriasis.^[15]^ Intravitreous corticosteroid injection is the major method for treating ocular posterior disorder; however, it may induce elevation of intraocular pressure, complicated cataract, secondary glaucoma, as well as endophthalmitis.^[16,17]^ In a previous study, periocular injection was superior to that of intravitreous injection in clinical safety. To be exact, periocular dexamethasone injection (5 mg) showed a 75-fold anti-inflammatory effect compared with that of cortocosteroids when reaching the vitreous body.^[18]^ This implicated that periocular corticosteroid injection is effective for treating ocular posterior diseases.

TA, a typical corticosteroid commonly used in clinical practice, shows 5-fold anti-inflammatory effects compared to that of the hydrocortisone. It involves in the anti-inflammation through modulating various inflammatory pathways, when then contributes to the stability of the blood–retinal barrier. Also, it may involve in the pathways against angiogenesis through attenuating the VEGF expression. It shows satisfactory intraocular tolerance, which is still active several months after reaching the vitreous space.^[9,19]^

To our best knowledge, intravitreous TA injection is the preferred method for treating neovascular age-related macular degeneration (AMD).^[20]^ Rare studies have been focusing on the treatment of PCV using periocular TA injection. In this study, 12 PCV cases with massive subretinal hemorrhage and exudation received periocular TA injection, in comparison to that of 10 cases received anti-VEGF therapy via intravitreous injection. In the 6-month follow-up, we determined the subretinal hemorrhage and exudation, visual acuity, and the OCT changes. None of the patients showed complications. The total efficiency for the TA injection was significantly higher than that of the anti-VEGF group (91.7% vs 60%). Compared with the pretreatment conditions, significant improvement was noticed in the visual acuity at month 1, 2, and 3 in TA group, respectively (*P* < .05). In contrast, no statistical differences were noticed in the anti-VEGF group at month 1 compared with the baseline level (*P* > .05), but significant improvement was noticed in the visual acuity at month 2 and 3, respectively (*P* < .05).

Despite anti-VEGF agents have been extensively used for treating AMD, the treatment efficiency is not satisfactory worldwide. Moreover, the treatment is expensive, which may bring a heavy load to the victims. These deserve more investigations on it. Although PCV may be a subtype of neovascular AMD,^[21]^ their pathogenesis may be different in nature. Maybe, inflammation plays an important role in the PCV rather than neovascular AMD. Whereas, VEGF is only a small part involving in the pathogenesis of inflammation. Therefore, the treatment of anti-VEGF therapy on PCV is not satisfactory. Interestingly, corticosteroids targeting various inflammatory mechanisms are proved to be effective for treating PCV.

We treated PCV using periocular TA injection in 10 cases with no massive subretinal hemorrhage and exudation before this study, but the efficiency was poor. We still cannot confirm why periocular TA injection is effective for treating PCV concurrent with HRD. In future, further studies are needed to discover the potential mechanisms. In addition, the sample size is small, which may hamper the accuracy of this study. However, it shows no effects on the amazing efficiency on the safety and feasibility in clinical practice.

In conclusion, periocular TA injection is effective for treating PCV concurrent with HRD. Its efficiency is more superior to that of intravitreal injection of anti-VEGF. Despite the sample size is small, we are really astonished by the amazing efficiency of the treatment regimen, as well as the safety and economic practicability. In future, large sample size clinical trials are needed to investigate the efficiency and safety of such method.

## Author contributions

**Data curation:** Xuyang Sun.

**Formal analysis:** Xuyang Sun, Qionglei Zhong.

**Funding acquisition:** Qionglei Zhong.

**Investigation:** Lin Lin.

**Methodology:** Lin Lin.

**Project administration:** Yuan Gao.

**Resources:** Yuan Gao.

**Software:** Fanlin Hong.

**Validation:** Fanlin Hong.

**Writing – original draft:** Kaiyan Zhang.

**Writing – review & editing:** Yingying Chen.
